# Analyzing Upper Extremity Disability From Health‐Related Quality of Life in Chronic Chikungunya: A Biopsychosocial Approach

**DOI:** 10.1111/jep.70590

**Published:** 2026-09-08

**Authors:** Raildo Oliveira da Silva Filho, Marynara Fabíola Silva Araújo, Andreia Ravinia do Nascimento Oliveira, Maria Eduarda Medeiros da Costa Figueiredo, Dimitri Taurino Guedes, Renata Newman Leite dos Santos Lucena, Leonildo Santos do Nascimento Junior, Thais Marques Lima, Eleazar Marinho de Freitas Lucena

**Affiliations:** ^1^ Universidade Federal do Rio Grande do Norte (UFRN) Santa Cruz Brazil; ^2^ Universidade Federal da Paraíba (UFPB) João Pessoa Brazil

**Keywords:** arthralgia, chikungunya virus, health‐related quality of life, upper extremity

## Abstract

**Relevance to the Journal:**

Responding to the Journal of Evaluation in Clinical Practice's emphasis on person‐centered care, this study examines how pain, functional capacity and vitality influence lived disability and rehabilitation needs in individuals with chronic chikungunya.

**Rationale:**

Chikungunya is an arbovirus transmitted by the *Aedes aegypti* and *Aedes albopictus* mosquitoes and is the second most prevalent pathogen related to acute febrile illnesses in Latin America. Among the three possible phases of the disease (acute, subacute, and chronic), the chronic phase occurs when symptoms persist for more than 90 days. During this period, many individuals may experience functional deficits in their upper limbs, compromising daily activities and affecting their quality of life.

**Aims and Objectives:**

The aim of this study was to analyze upper limb functional disability based on health‐related quality of life components in individuals with chronic chikungunya.

**Method:**

This cross‐sectional observational study, with a quantitative approach, was carried out in the municipality of Santa Cruz, Rio Grande do Norte, Brazil, from April to November 2023. Seventy‐four individuals with chronic chikungunya sequelae participated in the study and were assessed using three instruments: a sociodemographic and health status questionnaire, developed by the research team; the Short Form‐36 (SF‐36); and the Disabilities of the Arm, Shoulder and Hand (DASH) questionnaire. Data were analyzed using descriptive statistics and multiple linear regression to examine the independent association between SF‐36 domains and upper limb disability, adopting a significance level of 5%.

**Results:**

Pain (*β* = −0.410), functional capacity (*β* = −0.274) and vitality (*β* = −0.233) were independently associated with upper‐limb disability. Together, these variables explained 52.3% of the variability in DASH scores (*R* ^2^ = 0.523).

**Conclusion:**

Pain, functional capacity and vitality were independently associated with upper limb disability in individuals with chronic chikungunya. These findings support a person‐centered approach to rehabilitation by highlighting key domains that may guide individualized assessment and rehabilitation strategies for people living with chronic chikungunya.

## 1 Introduction

Chikungunya is a compulsory notifiable arbovirus transmitted by the Aedes aegypti and Aedes albopictus mosquitoes, the same vectors that transmit dengue and yellow fever [1]. It is considered the second most prevalent pathogen related to acute febrile illnesses in Latin America, behind only the dengue virus and ahead of the Zika virus [2].

The first autochthonous cases were recorded on the island of Saint Martin in December 2013. Since then, the chikungunya virus (CHIKV) has spread rapidly, causing epidemics throughout the American continent. Between December 2013 and June 2023, 3,684,554 cases (suspected and confirmed) were reported in 50 countries or territories in the Americas [3].

In the South American context, Brazil is the South American country with the highest number of cases [4]. Between March 2013 and June 2022, there were 253,545 cases confirmed by laboratory tests, distributed across 3,316 (59.5%) of Brazil's 5,570 municipalities. Since 2016, the country has been considered the epicenter of the epidemic in the Americas [3], having faced seven major epidemic waves between 2016 and 2022, with annual peaks ranging from 24,097 to 44,604 cases [5].

The state of Rio Grande do Norte (RN) recorded three major outbreaks of chikungunya fever—in early 2015, early 2016, and mid‐2019—and is one of the states with the highest incidence of the disease [6]. More recently, in the first half of 2023, the state had the third‐highest positivity rate in the country (37.4%), behind only Minas Gerais (50%) and Pará (43.1%) [7].

The main symptoms and characteristics of chikungunya are the triad: rash, fever, and intense pain in various joints of the body, while secondary complaints include headache, myalgia, and retro‐orbital pain [8].

The disease can manifest in up to three successive phases: acute, subacute, and chronic. The chronic phase is characterized by symptoms that persist for more than 90 days [9]. It is reported that the chronicity of the disease can affect 25%–50% of those affected [10, 11], and the main risk factors associated with this process are: age over 45 years; pre‐existing joint problems; and greater intensity of joint involvement during the acute phase [9]. Arthralgia remains the main sequela in those who progress to the last phase of the disease and is present in approximately 90% of cases [12].

Pain has been reported by patients throughout the day, and joint limitations directly and negatively interfere with the performance of daily activities, which worsens the quality of life of chronic patients [13]. A study that sought to investigate the correlation between the duration of infection and the level of joint pain and whether there was a decrease in functional capacity showed that for each month of infection, there was an increase in the level of pain, as well as a reduction in functional capacity, in addition to fear of performing common movements of daily living [14].

From a broader perspective, the impact of chikungunya cannot be fully understood without considering its social context. Evidence indicates that women and low‐income individuals are disproportionately affected, reflecting the role of social determinants of health, such as income and access to health services [15]. The biopsychosocial model is a useful framework for interpreting functional disability, integrating biological impairment (pain, inflammation [14]), psychological consequences (vitality, emotional well‐being [14]), and social vulnerability (living and working conditions [16]). This approach brings the study of functional disability in chikungunya closer to the field of public health.

Regarding upper limb involvement, one study associated chronic chikungunya with weakness in wrist movement and increased pain in the hands when a group of women with the disease was compared to a control group. Associated with these findings, there was impairment in performing daily tasks, such as placing objects above the head and carrying heavy bags [17].

The existence of upper limb disability in people affected by the chikungunya virus, especially in the chronic phase, still needs further investigation through observational studies that relate and quantify the factors involved in this process.

The present study not only describes upper limb disability in chronic chikungunya but also evaluates clinical determinants that may guide rehabilitation practice. By identifying which quality of life domains best predict DASH scores, the analysis provides evidence that can assist clinicians in developing targeted therapeutic strategies and decision‐making processes in both primary and specialized care settings.

Given this gap, the present study aimed to analyze upper limb functional disability in individuals with chronic chikungunya, considering components of quality of life through multivariate regression analysis.

## 2 Methods

### 2.1 Research Characteristics

This is an observational, cross‐sectional study with a quantitative approach, conducted in the municipality of Santa Cruz, Rio Grande do Norte, Brazil, involving individuals with chronic sequelae resulting from chikungunya.

### 2.2 Sampling Plan

The participants were recruited through social media posts (Instagram and Facebook). In addition, posters containing information about the study were displayed on bulletin boards and walls throughout the university's academic spaces. All promotional materials included a brief description of the study, the eligibility criteria, and the principal investigator's contact information, so that interested potential participants could get in touch and schedule an interview to assess their eligibility. Included were individuals with a medical report issued by a physician documenting a confirmed diagnosis of chikungunya, aged between 18 and 75 years, and with symptoms persisting for more than 3 months. Laboratory test results were not required as an inclusion criterion; when available, they could be considered by the physician in confirming the diagnosis and issuing the medical report. Participants diagnosed with other rheumatic diseases that could interfere with the assessment of the study's outcomes were excluded.

The minimum sample size was based on Green's equation [18], cited by Field [19], which suggests that for a multiple regression model, the sample size should be: 50 + 8k, where k is the number of predictors. Considering the model proposed in this study, with three variables being statistically significant to explain the outcome, the equation [50 + 8(3)] was applied, resulting in a total of 74 participants.

### 2.3 Variables

To specifically assess upper limb function, the Disabilities of the Arm, Shoulder, and Hand (DASH) questionnaire was applied in its Portuguese version [20]. Comprising 30 items, the instrument was originally developed by the Institute for Work & Health (Toronto, Ontario), the Council of the Musculoskeletal Specialty Societies (COMSS), and the American Academy of Orthopaedic Surgeons (AAOS) [21]. Scores range from 0 to 100, where 0 represents no disability and 100 represents maximum disability [22].

Quality of life was assessed using the Short Form 36 Health Survey (SF‐36) questionnaire, which provides a profile of scores to measure the physical and mental health of a population over the last 4 weeks. The questionnaire consists of 36 items that assess eight components related to quality of life: functional capacity, physical aspects, pain, general health status, vitality, social aspects, emotional aspects, and mental health. The total score ranges from 0 to 100, with higher scores indicating better quality of life [23].

### 2.4 Data Collection

Data collection took place between April and November 2023 through in‐person interviews conducted at the FACISA/UFRN Teaching Clinic in a private setting, ensuring the privacy and confidentiality of participants' information. Initially, a structured questionnaire developed by the researchers was administered, consisting of three sections: (1) sociodemographic data, including name, sex, age, marital status, education level, household income, occupation, race, and information on leisure activities; (2) medical history, covering past medical history and family history; and (3) health status, covering information related to the diagnosis of chikungunya, time since diagnosis, symptoms, sequelae, and other relevant clinical aspects. In addition, body weight and height were measured by the research team using standardized procedures, and body mass index (BMI) was calculated as weight (kg) divided by height squared (m^2^).

Next, the participants completed the Disabilities of the Arm, Shoulder, and Hand (DASH) questionnaire, used to assess upper limb function, and the Medical Outcomes Study 36‐Item Short‐Form Health Survey (SF‐36), used to assess health‐related quality of life. The average duration of the interview was approximately 40 min per participant.

### 2.5 Data Analysis

The Statistical Package for the Social Sciences (SPSS) version 23 was used for data analysis. In the descriptive analysis, measures of central tendency and dispersion were considered for continuous variables, and absolute and relative frequencies for categorical variables.

Initially, simple linear regression analyses were performed to evaluate the association between the DASH score (dependent variable) and each of the eight SF‐36 domains. Domains showing statistically significant associations (*p* < 0.05) were entered into multiple linear regression models. The final model was selected based on statistical significance and the adequacy of model assumptions, including normality of residuals, absence of multicollinearity, and independence of residuals. Because the present study was conducted using data from a pre‐existing research database, sample adequacy for the final regression model was assessed according to Green's recommendation for multiple linear regression (*N* ≥ 50 + 8*k*, where *k* represents the number of predictors). Considering the three predictors retained in the final model, the minimum recommended sample size was 74 participants, which corresponded to the study sample.

In addition, a multiple linear regression analysis was performed to develop a model to verify which components related to quality of life explain upper limb disability in people with chronic chikungunya, considering a significance level of 5%.

To analyze environments in which several variables affect a single outcome, multiple linear regression is an appropriate statistical approach. The quality of the model fit was assessed using the *p*‐value, which indicates statistical significance, and the adjusted R^2^, which showed the degree to which the independent variables explain the variance of the dependent variable, adjusting the value for population generalization [24].

The Kolmogorov‐Smirnov test was used to assess the adherence of the residuals to the normal distribution, with the normality hypothesis being rejected if the p‐value was less than 0.05. The variance inflation factor (VIF) was used to assess multicollinearity; values above 5 or 10 indicate problems, suggesting the need for adjustments to the model to increase its accuracy [25].

The Durbin‐Watson statistic was used to assess autocorrelation of the residuals, indicating whether the errors in a model were correlated with each other, especially in relation to time [25].

ANOVA was used to assess whether the predictive power of the model is significantly greater than expected by chance, indicating whether the predictor variables, taken together, contribute significantly to predicting the criterion variable. Finally, the coefficients (*B*), as well as the standardized coefficients (*β*), indicate the relative strength of each independent variable on the dependent variable, allowing direct comparison between the effects of each predictor [24].

By integrating DASH scores with SF‐36 domains in a multivariate regression model, this study adopts a pragmatic analytic strategy that helps translate patient‐reported outcomes into clinically actionable indicators. This approach strengthens the applicability of the findings to rehabilitation planning and evaluation of care processes for individuals in the chronic stage of chikungunya.

### 2.6 Ethical Aspects

The study followed the ethical precepts of Resolution No. 466/12 of the National Health Council. Each participant signed a Free Consent Form before the interview.

This study was submitted to and approved by the Human Research Ethics Committee (CEP) of the Trairi Faculty of Health Sciences (FACISA)—Federal University of Rio Grande do Norte (UFRN), under Opinion No. 6,004,392 (CAAE: 67614523.2.0000.5568).

## 3 Results

A total of 74 participants took part in the study. Table 1 shows the sociodemographic characteristics and health conditions of the total sample included.

**Table 1:** Sociodemographic Characteristics and Health Conditions of Participants (*n* = 74).

	*n*	%		*n*	%
**Sex**			**Physical activity**		
Female	64	86.5	Yes	38	51.4
Male	10	13.5	No	36	48.6
**Age**			**Comorbidity**		
20 to 39 years	9	12.2	Systemic Arterial Hypertension	35	47.3
40 to 59 years	35	47.3	Osteoarthritis	22	29.7
60 years or more	30	40.5	Diabetes	11	14.9
**Stable union**			Osteoporosis/Osteopenia	8	10.8
Yes	44	59.5	**Time since diagnosis**		
No	30	40.5	More than 12 months	50	67.6
**Occupation/Daily activities**			Up to 12 months	24	32.4
Manual work	40	54.1	**Persistent complaints**		
Not manual work	12	16.2	Joint pain	74	100
None	21	28.4	Joint stiffness	57	77.0
**Income (in minimum wages)**			Muscle pain	42	56.8
Up to two	41	55.4	Back pain	42	56.8
More than two	9	12.2	Edema	36	48.6
Don't know/no answer	24	32.4	Generalized weakness	19	25.7
**Schooling**			Headache	13	17.6
No formal education	25	33.8	**Joints where pain persisted**		
Elementary	14	18.9	Shoulders	54	73.0
High school	24	32.4	Knees	53	71.6
Higher education	11	14.9	Ankles	48	64.9
**Race**			Hands and wrists	46	62.2
White	18	24.3	Feet	41	55.4
Black	3	4.1	Spine	38	51.4
Brown	28	37.8	Hips	32	43.2
No answer	25	33.8	Elbows	26	35.1
**Use of pain medication**			**DASH rating**		
Yes	32	43.2	Good	22	29.7
No	42	56.8	Fair	34	45.9
**BMI rating**			Severe functional disability	18	24.3
Obesity	43	58.1			
Overweight	21	28.4			
Normal weight	10	13.5			

*Source:* Prepared by the authors (2025).

The sample was predominantly composed of women (86.5%, *n* = 64), and most participants were between 40 and 59 years of age (47.3%, *n* = 35). Most individuals were manual workers (54.1%, *n* = 40), were in stable relationships (59.5%, *n* = 44), had an income of up to two minimum wages (55.4%, *n* = 41), and had a level of education classified as no formal education (33.8%, *n* = 25). The most prevalent race, self‐reported by participants, was brown (37.8%, *n* = 28); however, a large part of the sample did not answer this question (33.8%, *n* = 25).

Regarding the volunteers' health conditions, most participants reported practicing regular physical activity (51.4%, *n* = 38), whereas 58.1% (*n* = 43) were classified as obese according to their Body Mass Index (BMI). The most prevalent comorbidity was systemic arterial hypertension (47.3%, *n* = 35), and most did not frequently use medications for pain control or relief (56.8%, *n* = 42).

Most participants had been diagnosed with chikungunya more than 12 months previously (67.6%, *n* = 50) and reported persistent joint pain (100%, *n* = 74), joint stiffness (77.0%, *n* = 57), muscle pain (56.8%, *n* = 42) and spinal pain (56.8%, *n* = 42). Among the joints with persistent pain, the most affected were the shoulders (73.0%, *n* = 54), knees (71.6%, *n* = 53), ankles (64.9%, *n* = 48), and hands and wrists (62.2%, *n* = 46).

The results of the DASH questionnaire can be expressed in general terms for the study population, with classifications of: excellent (< 20 points), good (20 to 39 points), fair (40 to 60 points), and poor (severe functional disability; > 60 points), as shown in Table 1, or evaluated item by item (Table 2). In the study sample, 22 participants (29.7%) had good functional conditions, while the majority obtained fair results (34 people; 45.9%) and 18 had severe functional disability (24.3%).

**Table 2:** Frequency distribution of the categorical variables from the DASH questionnaire (*Disabilities of the arm, shoulder and hand*).

Difficulty	No and mild *n* (%)	Moderate *n* (%)	Severe and unable *n* (%)
**Items**			
1. Open a tight or new jar.	29 (39.2)	25 (33.8)	20 (27.0)
2. Write.	60 (81.1)	11 (14.8)	3 (4.1)
3. Turn a key.	65 (87.8)	6 (8.1)	3 (4.1)
4. Prepare a meal.	55 (74.3)	12 (16.2)	7 (9.5)
5. Push open a heavy door.	28 (37.8)	25 (33.8)	21 (28.4)
6. Place an object on a shelf above your head.	29 (39.2)	24 (32.4)	21 (28.4)
7. Do heavy household chores.	17 (23.0)	22 (29.7)	35 (47.3)
8. Garden or do yard work.	37 (50.0)	23 (31.1)	14 (18.9)
9. Make a bed.	50 (67.6)	16 (21.6)	8 (10.8)
10. Carry a shopping bag or briefcase.	35 (47.3)	21 (28.4)	18 (24.3)
11. Carry a heavy object.	28 (37.8)	18 (24.4)	28 (37.8)
12. Change a lightbulb overhead.	26 (35.1)	18 (24.3)	30 (40.6)
13. Wash or blow dry your hair.	42 (56.8)	14 (18.9)	18 (24.3)
14. Wash your back.	40 (54.1)	14 (18.9)	20 (27.0)
15. Put a pullover sweater.	43 (58.1)	24 (32.4)	7 (9.5)
16. Use a knife to cut food.	49 (66.2)	14 (18.9)	11 (14.9)
17. Recreational activities which require little effort.	56 (75.7)	10 (13.5)	8 (10.8)
18. Recreational activities in which you take some force or impact through your arm, shoulder or hand.	26 (35.1)	21 (28.4)	27 (36.5)
19. Recreational activities in which you move your arm freely.	26 (35.1)	20 (27.1)	28 (37.8)
20. Manage transportation needs (getting from one place to another).	45 (60.8)	15 (20.3)	14 (18.9)
21. Sexual activities.	56 (75.7)	10 (13.5)	8 (10.8)

Interference with social activities	Not at all and slightly *n* (%)	Moderately *n* (%)	Quite a bit and extremely *n* (%)
22. During the past week, to what extent has your arm, shoulder or hand problem interfered with your normal social activities with family, friends, neighbors or groups?	44 (59.4)	13 (17.6)	17 (23.0)

Limitations in work and daily activities	Not limited at all and slightly *n* (%)	Moderately *n* (%)	Very and unable *n* (%)
23. During the past week, were you limited in your work or other regular daily activities as a result of your arm, shoulder or hand problem?	38 (51.4)	18 (24.3)	18 (24.3)

Severity of symptoms in the last week	None and mild *n* (%)	Moderate *n* (%)	Severe and extreme *n* (%)
24. Arm, shoulder or hand pain.	24 (32.4)	31 (41.9)	19 (25.7)
25. Arm, shoulder or hand pain when you performed any specific activity.	24 (32.4)	31 (41.9)	19 (25.7)
26. Tingling (pins and needles) in your arm, shoulder or hand.	30 (40.6)	22 (29.7)	22 (29.7)
27. Weakness in your arm, shoulder or hand.	46 (62.2)	21(28.4)	7 (9.4)
28. Stiffness in your arm, shoulder or hand.	46 (62.2)	18 (24.3)	10 (13.5)

Difficulty sleeping	No and mild *n* (%)	Moderate *n* (%)	Severe and so much difficulty that I can't sleep *n* (%)
29. During the past week, how much difficulty have you had sleeping because of the pain in your arm, shoulder or hand?	40 (54.1)	21 (28.4)	13 (17.5)

Self‐perception of health status	Strongly disagree and disagree *n* (%)	Neither agree nor disagree *n* (%)	Agree and strongly agree *n* (%)
30. I feel less capable, less confident or less useful because of my arm, shoulder or hand problem.	34 (45.9)	10 (13.5)	30 (40.6)

*Source:* Prepared by the authors (2025).

Among the items evaluated in the questionnaire, the most compromised activities included those with severe difficulty or even inability to perform, notably: “doing heavy housework” (47.3%, *n* = 35), “changing a light bulb above one's head” (40.6%, *n* = 30), ‘carrying a heavy object' (37.8%, *n* = 28), and “recreational activities in which one moves one's arm freely” (37.8%, *n* = 28).

In addition to the specific activities mentioned, many participants reported significant interference in social activities, with 23% of participants (*n* = 17) indicating that their normal interactions with family, friends, neighbors, or colleagues were “quite” or “extremely” affected. As for work or daily activities, almost a quarter of the volunteers (24.3%, *n* = 18) reported that problems with their arm, shoulder, or hand made it difficult to perform everyday tasks. In addition, an equivalent proportion (24.3%, *n* = 18) reported moderate impairment.

With regard to severe or extreme physical symptoms perceived during the week prior to the questionnaire, skin discomfort (prickling sensation) in the arm, shoulder, or hand was the most prevalent, accounting for 29.7% of complaints (*n* = 22). Severe difficulty or inability to sleep was present in 17.5% of individuals (*n* = 13), with moderate intensity in 28.4% (*n* = 21). Finally, regarding self‐perceived health, 40.6% of study participants (*n* = 30) reported feeling less capable, less confident, and less useful due to the problem with their arm, shoulder, or hand.

The SF‐36 generic quality of life scale (Table 3) ranges from 0 to 100, where 0 indicates the worst state and 100 indicates the best state for each domain. The results showed greater impairment in the following components: physical limitations, pain, emotional limitations, and functional capacity, all with average scores below 50. In addition, “general health status” and “vitality” also had low scores. Only the components “social aspects” and “mental health” achieved values above 65.

**Table 3:** Measures of Central Tendency and Dispersion for the Quantitative Variables from the SF‐36.

Components	Mean	95% CI
Functional capacity	46.9	41.5; 52.2
Limitation due to physical aspects	30.4	21.6; 39.1
Pain	43.1	39.3; 46.9
General state of health	51.4	46.8; 55.9
Vitality	53.7	48.5; 59.0
Social aspects	66.7	60.2; 73.1
Limitation due to emotional aspects	46.4	36.0; 56.7
Mental health	68.4	63.3; 73.4

*Source:* Prepared by the authors (2025).

The final multiple linear regression model (Table 4) presented an *R* ^2^ of 0.523, with satisfactory fit measures. The Kolmogorov‐Smirnov test indicated the normality of the residuals (*p*‐value = 0.358). The variance inflation factor (VIF), inversely proportional to tolerance, measures multicollinearity among independent variables. The VIF measures of the predictor variables were all less than 5 (pain: 1.406; functional capacity: 1.550; and vitality: 1.205), confirming the absence of multicollinearity. The Durbin‐Watson statistic, used to verify the independence of the residuals, was 2.195, indicating the absence of residual correlation.

**Table 4:** Summary of the statistical measures of the estimated linear regression model.

Model	*R*	*R* ^*2*^	Adjusted *R* ^2^	Standard error of the estimate	Durbin‐Watson
1	0.723	*0.*523	0.502	10.41595	2.195

	Sum of squares	*Degrees of freedom*	Degrees of freedom	*F*	*p*
Regression	8324.727	3	2774.909	25.577	< 0.001
Residual	7594.434	70	108.492		
Total	15919.161	73			

Predictors	Coefficients (B)	95% CI for B	Standardized coefficients (β)	*t*	*p*
Constant	81.611	73.605; 89.617		20.330	< 0.001
Pain	−0.370	−0.545; −0.194	−0.410	−4.192	< 0.001
Functional capacity	−0.175	−0.305; −0.044	−0.274	−2.662	0.010
Vitality	−0.152	−0.270; −0.034	−0.233	−2.575	0.012

*Source:* Prepared by the authors (2025).

With regard to the independent variables, the “pain” component presented a negative and statistically significant standardized coefficient (*β* = −0.410; *p* < 0.001), indicating that an increase in pain score is associated with a reduction in the DASH score, i.e., less upper limb disability. The variable “functional capacity” also showed a negative correlation (*β* = −0.274; *p* = 0.010), suggesting that higher functional capacity scores are associated with lower DASH scores and, consequently, less upper limb disability. Similarly, higher vitality scores (*β* = −0.233; *p* = 0.012) were significantly associated with lower DASH scores, indicating better upper limb function.

These results suggest that quality of life components such as “pain,” “functional capacity,” and “vitality” significantly predict upper limb disability (DASH score), explaining approximately 52.3% of the variability (F3,70 = 25.577, *p* < 0.001) in this sample of patients in the chronic phase of chikungunya.

## 4 Discussion

The sample profile highlights the influence of social determinants of health. These characteristics suggest accumulated social vulnerabilities: a higher risk of chronic symptoms combined with fewer resources to deal with them. Manual work often requires repetitive physical effort, which may be substantially limited by upper limb functional impairment, while low education and income can restrict access to adequate care and rehabilitation services, perpetuating the cycle between illness and social disadvantage [15, 16]. These vulnerabilities may contribute to a greater functional burden associated with persistent musculoskeletal symptoms [15, 16].

The high prevalence of persistent musculoskeletal symptoms observed in this study reinforces the substantial burden of chronic chikungunya on affected individuals. These findings are consistent with those reported in a cross‐sectional study conducted in Amapá, Brazil, which identified a high prevalence of muscle pain (75.4%) and back pain (57.3%) among individuals infected with CHIKV, highlighting the persistence of musculoskeletal manifestations during the chronic phase of the disease [26]. Persistent pain, joint stiffness, and myalgia may be explained by a sustained inflammatory response triggered by CHIKV infection, leading to prolonged joint and muscle damage that can persist for months or even years after the acute phase.

The functional repercussions of these symptoms were assessed using the DASH questionnaire, a widely used instrument for evaluating upper limb function in different clinical conditions [20]. The findings indicate that chronic chikungunya substantially compromises upper limb function, particularly in activities requiring greater muscle strength and overhead movements. These results are consistent with previous studies reporting that persistent pain, joint stiffness, and reduced muscle strength continue to impair upper limb function during the chronic phase of the disease [26, 27]. Together, these findings reinforce the importance of rehabilitation strategies aimed at minimizing functional limitations and improving the performance of daily activities in individuals with chronic chikungunya.

At the same time, quality of life assessed by the SF‐36 showed greater impairment in the domains of functional capacity, physical aspects, pain, and emotional aspects. In addition, the domains of general health status and vitality also had lower scores, while social aspects and mental health achieved higher values. These results corroborate studies that assessed quality of life in individuals in the chronic phase of chikungunya, showing greater impairment in physical domains, especially in functional capacity and pain [28]. In this scenario of physical limitation and pain, physical activity emerges as a relevant variable to consider.

Although most studies have consistently reported substantial impairment in the physical domains among individuals with chronic chikungunya, the magnitude of these impairments has varied across studies. For example, while our participants showed relatively preserved scores for social aspects and mental health, other investigations have reported broader impairment across additional quality of life domains [29]. This variability may reflect differences in disease duration, clinical severity, sample characteristics, access to rehabilitation, and the instruments used to assess quality of life and functional disability. These methodological differences should be considered when comparing findings across studies [29].

Despite these physical limitations, most participants reported engaging in regular physical activity, although obesity remained highly prevalent. While these variables were not included in the regression model, regular physical activity has been recognized as an important non‐pharmacological strategy in the management of chronic chikungunya, with potential benefits for functional capacity and quality of life [22, 26, 30].

This perspective is consistent with the results of the multiple linear regression model, which identified pain, functional capacity, and vitality as independent predictors of upper limb disability. Among these variables, pain showed the strongest association with disability, reinforcing its central role in limiting upper limb function in individuals with chronic chikungunya. Functional capacity also emerged as an important predictor, suggesting that interventions aimed at improving physical performance may contribute to reducing disability. In addition, the association between vitality and upper limb function highlights the importance of considering energy and fatigue, aspects that have received less attention in previous studies but may directly influence daily functioning and engagement in rehabilitation. Taken together, these findings reinforce the multifactorial nature of disability, indicating that upper limb dysfunction in chronic chikungunya cannot be explained solely by biological impairment, but also involves broader dimensions of health‐related quality of life.

Although the proposed model explained approximately 52% of the variability in upper limb disability, nearly half of the variance remained unexplained. This finding suggests that additional biological, psychological, behavioral, and contextual factors not evaluated in the present study may also contribute to functional disability in chronic chikungunya.

Interpreting these findings under the biopsychosocial model highlights how persistent biological impairment (pain and stiffness) interacts with psychological aspects (reduced vitality, fear of movement) and social conditions (work demands, loss of income, limited access to health services). This perspective, while reinforcing findings from previous studies showing that chronic chikungunya reduces quality of life and functional capacity, emphasizes the intersection with social determinants and contributes to a more comprehensive understanding of the disease in the field of public health [16].

From a person‐centered care perspective, the integration of patient‐reported domains such as pain, functional capacity and vitality into clinical encounters may enhance shared decision‐making and support the definition of individualized rehabilitation goals. Addressing vitality and psychosocial dimensions in parallel with pain management can improve engagement, reduce fear of movement and potentially enhance functional outcomes among individuals living with chronic chikungunya.

Some limitations should be acknowledged. The cross‐sectional design precludes causal inference, and the convenience sample may limit the generalizability of the findings. Furthermore, although the regression model explained a substantial proportion of upper limb disability, other potentially relevant biopsychosocial factors were not evaluated and should be investigated in future prospective investigations. In addition, because the regression model was developed from variables selected after preliminary univariable analyses, its findings should be interpreted within the context of this exploratory observational study.

In this sense, rehabilitation programs that combine physical exercise, psychological support, and social interventions can be particularly effective in improving functional capacity and reducing pain. Evidence indicates that functional training programs, performed both on land and in an aquatic environment, promote significant pain reduction and improved functional capacity in patients affected by chikungunya [31].

Routine screening of pain, functional capacity and vitality using instruments such as the SF‐36 may help clinicians identify individuals with greater functional vulnerability [31]. In addition, simple and low‐cost interventions – including graded exercise, pain education and occupational guidance – should be further evaluated as feasible rehabilitation strategies for this population.

## 5 Conclusions

The present study demonstrated that upper limb disability in individuals with chronic chikungunya is strongly associated with the domains of pain, functional capacity, and vitality. Together, these three domains explained approximately 52.3% of the variability in upper limb disability, highlighting their relevance as key targets for clinical assessment and rehabilitation. Incorporating these domains into follow‐up protocols and local healthcare planning may facilitate early identification of individuals with greater functional vulnerability, support equitable access to rehabilitation services, and contribute to reducing the long‐term burden of disability in affected communities.

Additionally, the analysis points to the importance of integrated care strategies that consider not only biological dimensions but also psychosocial aspects and social inequalities that intensify the burden of the disease. Such a perspective can guide more equitable public policies and rehabilitation actions, contributing to improving the quality of life and well‐being of affected populations.

## Conflicts of Interest

The authors declare no conflicts of interest.

## Data Availability

The data that support the findings of this study are available on request from the corresponding author. The data are not publicly available due to privacy or ethical restrictions.

## References

[1] T. Y. V. de Lima Cavalcanti , M. R. Pereira , S. O. de Paula , and R. F. O. Franca , “A Review on Chikungunya Virus Epidemiology, Pathogenesis and Current Vaccine Development,” Viruses 14, no. 5 (2022): 969, 10.3390/v14050969. 35632709 PMC9147731

[2] J. Moreira , C. S. Bressan , P. Brasil , and A. M. Siqueira , “Epidemiology of Acute Febrile Illness in Latin America,” Clinical Microbiology and Infection 24 (2018): 827–835, 10.1016/j.cmi.2018.05.001. 29777926 PMC7172187

[3] W. M. Souza , G. S. Ribeiro , S. T. S. Lima , et al., “Chikungunya: A Decade of Burden in the Americas,” Lancet Reg Saúde Am 8, no. 30 (2024): 100673, 10.1016/j.lana.2023.100673. PMC10820659 38283942

[4] Pan American Health Organization , Epidemiological update for Dengue, Chikungunya and Zika in 2022 [Internet] (Washington: PAHO, 2022), https://www3.paho.org/data/index.php/en/mnu-topics/indicadores-dengue-en/annual-arbovirus-bulletin-2022.html.

[5] W. M. De Souza , S. T. S. de Lima , L. M. Simões Mello , et al., “Spatiotemporal Dynamics and Recurrence of Chikungunya Virus in Brazil: An Epidemiological Study,” Lancet Microbe 4, no. 5 (2023): e319–e329, 10.1016/S2666-5247(23)00033-2. 37031687 PMC10281060

[6] J. Xavier , V. Fonseca , J. F. Bezerra , et al., “Chikungunya Virus ECSA Lineage Reintroduction in the Northeasternmost Region of Brazil,” International Journal of Infectious Diseases 105 (2021): 120–123, 10.1016/j.ijid.2021.01.026. 33476757

[7] Brasil. Ministério da Saúde ., “Secretaria de Vigilância em Saúde. Monitoramento Dos Casos De Arboviroses Urbanas Causadas Por Vírus Transmitidos Pelo Mosquito Aedes (Dengue, Chikungunya E Zika), Semanas Epidemiológicas 1 a 35,” Boletin Epidemiologico 54, no. 13 (2023): 1–24, https://www.gov.br/saude/pt-br/centrais-de-conteudo/publicacoes/boletins/epidemiologicos/edicoes/2023/boletim-epidemiologico-volume-54-no-13/view.

[8] K. Bartholomeeusen , M. Daniel , D. A. LaBeaud , et al., “Chikungunya Fever,” Nature Reviews Disease Primers 9 (2023): 17, 10.1038/s41572-023-00429-2. PMC11126297 37024497

[9] Brasil ., “Ministério da Saúde. Secretaria de Vigilância em Saúde,” Departamento de Vigilância das Doenças Transmissíveis. Chikungunya: Manejo Clínico. Brasília: Ministério da Saúde (2017): 65, https://bvsms.saude.gov.br/bvs/publicacoes/chikungunya_manejo_clinico.pdf.

[10] J. K. Amaral , J. B. Bilsborrow , and R. T. Schoen , “Brief Report: The Disability of Chronic Chikungunya Arthritis,” Clinical Rheumatology 38, no. 7 (2019): 2011–2014, 10.1007/s10067-019-04529-x. 30963336 PMC7400963

[11] C. S. Lázari , M. S. Ramundo , F. ten‐Caten , et al., “Clinical Markers of Post‐Chikungunya Chronic Inflammatory Joint Disease: A Brazilian Cohort,” PLoS Neglected Tropical Diseases 17, no. 1 (2023): e0011037, 10.1371/journal.pntd.0011037. 36608155 PMC9851532

[12] J. F. Lemos , L. M. C. Araújo , V. J. G. Carmo , et al., “Prevalência, Articulações Acometidas E Intensidade Das Artralgias Em Indivíduos Na Fase Crônica Da Febre Chikungunya,” Brazilian Journal of Pain 4, no. 2 (2021): 108–112, 10.5935/2595-0118.20210032.

[13] T. H. P. Santos , M. P. Amaral , D. R. M. Brasil , et al., “Symptomatic Perception of Patients Affected by Chronic Chikungunya: A Qualitative Perspective,” Journal of Biological and Health Sciences 10, no. 1 (2022): 1–5, 10.12662/2317-3206jhbs.v10i1.4604.p1-5.2022.

[14] C. G. Souza , J. F. Costa , D. Dantas , et al., “Evaluation of Pain, Functional Capacity and Kinesiophobia in Women in the Chronic Stage of Chikungunya Virus Infection: A Cross‐Sectional Study in Northeastern Brazil,” Acta Tropica 199 (2018): 105118, 10.1016/j.actatropica.2018.12.008. 30529444

[15] F. A. V. Couceiro , F. K. M. Furtado , G. S. Guedes , et al., “Epidemiology of Chikungunya in Brazil: Socioeconomic and Health Context Between 2017 and 2021,” Research, Society and Development 11, no. 7 (2022): e46611730331, 10.33448/rsd-v11i7.30331.

[16] A. F. L. Cavalcante , A. Okano , M. T. Micussi , et al., “Artralgia Crônica Por Chikungunya Reduz Funcionalidade, Qualidade De Vida E Performance Ocupacional: Estudo Descritivo Transversal,” Brazilian Journal of Pain 5, no. 3 (2022): 233–238, 10.5935/2595-0118.20220047-pt.

[17] G. L. R. Machado , R. Q. Castro , L. Forechi , H. C. Souza , D. S. Fonseca , and M. A. C. Garcia , “The Impact of Chikungunya Chronic Arthralgia on Women's Upper Limbs Motor Function: A Cross‐Sectional Study,” Fisioterapia e Pesquisa 29, no. 4 (2022): 412–420, 10.1590/1809-2950/22011229042022EN.

[18] S. B. Green , “How Many Subjects Does It Take to Do a Regression Analysis?,” Multivariate Behavioral Research 26, no. 3 (1991): 499–510. 26776715 10.1207/s15327906mbr2603_7

[19] A. Field Descobrindo a Estatística Usando o SPSS. 2^a^ ed. Porto Alegre: Artmed; 2009.

[20] A. G. Orfale , P. M. P. Araújo , M. B. Ferraz , and J. Natour , “Translation into Brazilian Portuguese, Cultural Adaptation and Evaluation of the Reliability of the Disabilities of the Arm, Shoulder and Hand Questionnaire,” Brazilian Journal of Medical and Biological Research 38, no. 2 (2005): 293–302, 10.1590/S0100-879X2005000200018. 15785841

[21] P. L. Hudak , P. C. Amadio , and C. Bombardier , “Development of An Upper Extremity Outcome Measure: The Dash (Disabilities of the Arm, Shoulder and Hand) [Corrected]. The Upper Extremity Collaborative Group (UECG),” American Journal of Industrial Medicine 29, no. 6 (1996): 602–608, 10.1002/(SICI)1097-0274(199606)29:6<602::AID-AJIM4>3.0.CO;2-L. 8773720

[22] I. L. Neumann , D. A. de OLIVEIRA , E. L. de BARROS , et al., “Resistance Exercises Improve Physical Function in Chronic Chikungunya Fever Patients: A Randomized Controlled Trial,” European Journal of Physical and Rehabilitation Medicine 57, no. 4 (2021): 620–629, 10.23736/S1973-9087.21.06520-5. 33448754

[23] R. M. Ciconelli , “Tradução Para Língua Portuguesa E Validação do Questionário Genérico de Avaliação de Qualidade de Vida SF‐36 (Brasil SF‐36),” Revista Brasileira de Reumatologia 39, no. 3 (1999): 143–150, https://tosaudefuncional.com/wp-content/uploads/2013/03/questionc3a1rio-de-qualidade-de-vida-sf36-traduc3a7c3a3o-e-validac3a7c3a3o.pdf.

[24] C. P. Dancey , J. G. Reidy , and R. Rowe , “Estatística Sem Matemática Para Ciências da Saúde.” Tradução técnica: Lori Viali (Porto Alegre: Penso: (2017).

[25] J. F. Hair , W. C. Black , B. J. Babin , et al., “Análise multivariada de dados [recurso eletrônico].” Tradução: Adonai Schlup Sant'Anna (Bookman, 2009). 6^a^ ed, https://ia903108.us.archive.org/33/items/kupdf.net_hair-j-f-anaacutelise-multivariada-de-dados-6ordf-ediccedilatildeopdf/kupdf.net_hair-j-f-anaacutelise-multivariada-de-dados-6ordf-ediccedilatildeopdf.pdf.

[26] J. B. O. Silva , L. S. M. Júnior , V. F. S. Tavares , et al., “Correlação Entre a Resistência Cardiorrespiratória E a Qualidade De Vida Em Indivíduos Com Sintomas Crônicos Da Infecção Pelo Vírus Chikungunya,” Revista Brasileira de Qualidade de Vida 14 (2022): e14774, 10.3895/rbqv.v14.14774.

[27] A. F. L. Cavalcante , Artralgia Crônica Após a Febre Chikungunya: Estudo Transversal do Perfil Funcional e Laboral [Dissertação] (Santa Cruz (RN): Universidade Federal do Rio Grande do Norte, Faculdade de Ciências da Saúde do Trairi, 2020).

[28] A. C. B. Santos , “Avaliação Da Qualidade De Vida Em Indivíduos No Estágio Crônico Da Infecção Pelo Vírus Chikungunya [Trabalho De Conclusão De Curso],” Vitória de Santo Antão (PE): Universidade Federal de Pernambuco, Centro Acadêmico de Vitória (2019), https://repositorio.ufpe.br/handle/123456789/32325.

[29] G. Tiozzo , A. M. de Roo , G. S. Gurgel do Amaral , H. Hofstra , G. T. Vondeling , and M. J. Postma , “Assessing Chikungunya's Economic Burden and Impact on Health‐Related Quality of Life: Two Systematic Literature Reviews,” PLoS Neglected Tropical Diseases 19, no. 5 (2025): e0012990, 10.1371/journal.pntd.0012990. 40323984 PMC12074603

[30] M. A. Rios , B. T. Goes , L. S. R. Oliveira , et al., “Exercícios Físicos No Controle De Dor Ou Fadiga Associadas Às Infecções Virais: Revisão Sistemática,” Brazilian Journal of Pain 5, no. 3 (2022), 10.5935/2595-0118.20220048-pt.

[31] Y. A. Almeida , A. R. L. Santos , A. K. F. Silva , et al., “Relação Da Dor E Limitações Funcionais Em Pessoas na Fase Crônica da Chikungunya,” Estação Científica 17, no. 30 (2023), https://estacio.periodicoscientificos.com.br/index.php/estacaocientifica/article/view/2725.

